# A Distinguished Roadmap of Fibroblast Senescence in Predicting Immunotherapy Response and Prognosis Across Human Cancers

**DOI:** 10.1002/advs.202406624

**Published:** 2024-12-30

**Authors:** Dongjie Chen, Pengyi Liu, Jiayu Lin, Longjun Zang, Yihao Liu, Shuyu Zhai, Xiongxiong Lu, Yuanchi Weng, Hongzhe Li

**Affiliations:** ^1^ Department of General Surgery Pancreatic Disease Center Ruijin Hospital Shanghai Jiao Tong University School of Medicine Shanghai 200025 China; ^2^ Research Institute of Pancreatic Diseases Shanghai Key Laboratory of Translational Research for Pancreatic Neoplasms Shanghai Jiao Tong University School of Medicine Shanghai 200025 China; ^3^ State Key Laboratory of Oncogenes and Related Genes Institute of Translational Medicine Shanghai Jiao Tong University Shanghai 200025 China; ^4^ Department of General Surgery Taiyuan Central Hospital Taiyuan Shanxi 030009 China

**Keywords:** fibroblast senescence, immunotherapy, pan‐cancer analysis, scRNA‐seq, survival prognostication

## Abstract

The resistance of tumors to immune checkpoint inhibitors (ICI) may be intricately linked to cellular senescence, although definitive clinical validation remains elusive. In this study, comprehensive pan‐cancer scRNA‐seq analyses identify fibroblasts as exhibiting the most pronounced levels of cellular senescence among tumor‐associated cell populations. To elucidate this phenomenon, a fibroblast senescence‐associated transcriptomic signature (FSS), which correlated strongly with protumorigenic signaling pathways and immune dysregulation that fosters tumor progression, is developed. Leveraging the FSS, the machine learning (ML) framework demonstrates exceptional accuracy in predicting ICI response and survival outcomes, achieving superior area under curve (AUC) values across validation, testing, and in‐house cohorts. Strikingly, FSS consistently outperforms established signatures in predictive robustness across diverse cancer subtypes. From an integrative analysis of 17 CRISPR/Cas9 libraries, CDC6 emerges as a pivotal biomarker for pan‐cancer ICI response and prognostic stratification. Mechanistically, experimental evidence reveals that CDC6 in tumor cells orchestrates fibroblast senescence via TGF‐β1 secretion and oxidative stress, subsequently reprogramming the tumor microenvironment and modulating ICI response. These findings underscore the translational potential of targeting fibroblast senescence as a novel therapeutic strategy to mitigate immune resistance and enhance antitumor efficacy.

## Graphical abstract

This study investigates the link between cellular senescence and resistance to immune checkpoint inhibitors (ICI) in cancer. Through pan‐cancer scRNA‐seq, fibroblasts are identified as key drivers of senescence, with a novel fibroblast senescence‐associated signature outperforming existing models in predicting ICI response. Experimental insights highlight CDC6 as a pivotal regulator, offering translational strategies to improve antitumor immunity.

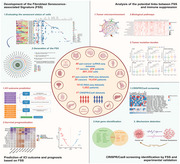

## 1 Introduction

Cellular senescence represents a stable cessation of the cell cycle, occurring in diploid cells and constraining their proliferative capacity. It serves as a protective mechanism against genomic instability, thus mitigating the accumulation of DNA damage.^[^ ^1^, ^2^ ^]^ Oncogenic stress induced by aberrant expression of certain oncogenes or loss of tumor suppressor genes can also elicit senescence in tumor cells. Initially, senescence serves as a barrier to tumor progression which highlights its potential as an anticancer mechanism.^[^ ^3^ ^]^ However, evidence is mounting to suggest that this cellular condition triggers pro‐tumorigenic impacts, such as fostering the proliferation of cancer cells, promoting angiogenesis, and impeding tumor immunity.^[^ ^4^, ^5^, ^6^ ^]^ Given that senescence has been acknowledged as a hallmark of cancer recently,^[^ ^7^ ^]^ the identification of senescent cells stands as a crucial measure in averting any protumorigenic occurrences and holding promise for the personalized therapy of cancer.

As a key stromal element within the tumor microenvironment (TME), cancer‐associated fibroblasts (CAFs) exhibit notable biological diversity across various aspects, encompassing their cellular origin, phenotype, and functional attributes.^[^ ^8^, ^9^ ^]^ Extensive prior literature has underscored the engagement of CAFs across multiple stages of tumorigenesis through varied molecular pathways.^[^ ^10^ ^]^ By engaging in reciprocal signaling cascades with tumor cells and neighboring cellular constituents via CAF‐secreted cytokines, chemokines, growth factors, and exosomes within the TME, CAFs not only bolster tumor proliferation but also orchestrate immune evasion mechanisms employed by cancer cells.^[^ ^11^, ^12^ ^]^ Furthermore, CAFs possess the capacity to remodel the stromal extracellular matrix (ECM) via the release of matrix metalloproteinases (MMPs) alongside the synthesis of novel matrix constituents, thus fostering structural support for tumor invasion and angiogenesis.^[^ ^13^ ^]^ Plentiful evidence also suggests that senescent fibroblasts have the potential to foster tumorigenesis and the expansion of tumors across various cancers.^[^ ^14^, ^15^, ^16^, ^17^ ^]^ In essence, the precise functional roles and intricate mechanistic underpinnings of CAFs in cancer pathogenesis and immunotherapy warrant further elucidation.

Forecasting individual survival and anticipating therapy response is pivotal in crafting personalized treatment strategies within precision oncology. Considering the pivotal function of the CAFs in tumorigenesis, we proposed that the senescent CAFs significantly contribute to tumor advancement and affect the effectiveness of immunotherapy in solid tumors. Therefore, targeting fibroblast senescence holds promise for predicting survival outcomes and responses to immunotherapy.

**Figure 1:**
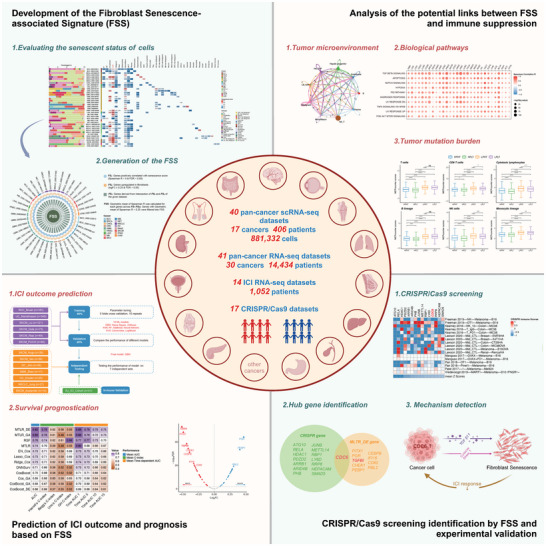
The workflow of this study.

In our research, we initially characterized the senescent state of CAFs in 40 pan‐cancer scRNA‐seq datasets (comprising 17 types of cancer, 406 patients, and 881332 cells). Subsequently, we established a fibroblast senescence‐associated signature (FSS) based on these large‐scale scRNA‐seq datasets. The predictive efficacy of FSS in predicting immune checkpoint inhibitor (ICI) response was further evaluated and confirmed using 10 machine learning (ML) algorithms across 14 pan‐cancer bulk ICI RNA‐seq datasets, encompassing 1052 patients. Additionally, we employed 15 ML algorithms specific to survival analysis as part of the SurvBenchmark^[^ ^18^ ^]^ framework to assess the survival predictive power of FSS, involving an integrated examination of RNA‐seq data from The Cancer Genome Atlas (TCGA) spanning 30 cancer types. Finally, CDC6 emerged as the most promising target within FSS, as determined through analysis of 17 CRISPR/Cas9 cohorts, and its efficacy in pancreatic cancer (PC) was validated through experimental investigation. The graphical abstract of this study is depicted in **Figure** **1**.

## 2 Results

### 2.1 Senescence Atlas of Cells in TME

**Figure 2:**
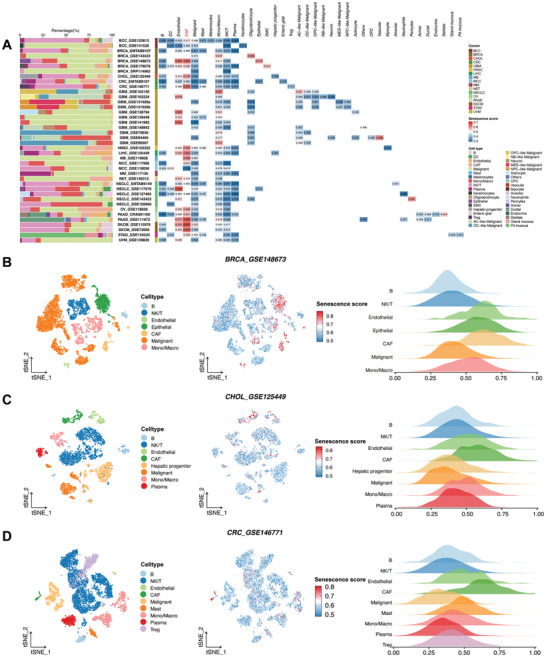
Evaluating the senescent status of individual cells in 40 pan‐cancer scRNA‐seq datasets. A) Left panel: The proportion of annotated cells among 40 pan‐cancer scRNA‐seq datasets. Right panel: Heatmap illustrating the distribution of senescence score among 40 pan‐cancer scRNA‐seq datasets. B–D, left) t‐distributed stochastic neighbor embedding (t‐SNE) plots showing the cell type annotations from 3 scRNAseq datasets. B–D, middle) Feature plots showing the distribution of senescence scores per cell in colors. B–D, right) Ridge plots showing the distribution of the senescence scores across cell clusters.

In this study, we proposed that transcriptomic alterations associated with the senescence of CAFs could serve as a robust biomarker for predicting both survival outcomes and response to ICI in cancer patients. To substantiate this hypothesis, we analyzed 40 pan‐cancer scRNA‐seq datasets encompassing 881332 cells, after isolating and clustering high‐quality cells (Table S1, Supporting Information). Using a curated gene set from the CellAge database (https://genomics.senescence.info/cells) as a metric for cellular senescence, Gene Set Variation Analysis (GSVA) revealed that CAFs exhibited the highest GSVA scores (referred to as senescence scores, min‐max normalization) across 40 cancer types, surpassing malignant cells and other cellular populations within the TME, such as B cells, NK/T cells, and endothelial cells (**Figure** **2**A). Subgroup analyses of three specific cancer types—breast cancer (GSE148673), cholangiocarcinoma (GSE125449), and colorectal cancer (GSE146771)—further demonstrated elevated senescence scores in CAFs, visualized using t‐distributed stochastic neighbor embedding (t‐SNE) plots (Figure 2B–D).

Senescent cells are characterized by a distinct phenotype associated with the secretion of bioactive molecules capable of modulating the activation states of neighboring cells.^[^ ^19^ ^]^ To investigate the intercellular communication between senescent CAFs and other cell populations, CAFs were stratified into high‐senescent (HS‐CAF) and low‐senescent (LS‐CAF) subgroups based on the median senescence score derived above. Cell–cell communication analysis revealed that HS‐CAF exhibited a significantly higher degree of interaction within the TME compared to LS‐CAF, particularly with immune cell subsets (Figure S1A–C, Supporting Information). Collectively, our findings suggest that the senescence of CAFs not only enhances their interactions with immune cells but also plays a central role in shaping the dynamics of the TME.

### 2.2 Establishment of an FSS via Pan‐Cancer scRNA‐seq Analysis

**Figure 3:**
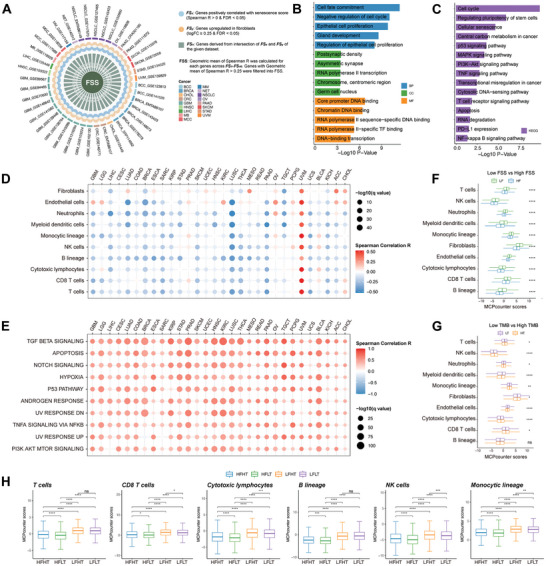
Establishment of the fibroblast senescent‐associated signature (FSS). A) Circos plot depicting the establishment of FSS. The outer circle represents the enrolled 40 pan‐cancer scRNA‐seq datasets. The middle circle represents the veen plot depicting the intersection of *FSx* (blue) and *FSy* (brown). Barplot illustrating the pathway enrichment analysis based on GO B) and KEGG C) databases. D) Dotplot depicting the correlation between FSS and the infiltration of immune cells across diverse cancer types. E) Dotplot depicting the correlation between FSS and the top 10 hallmark pathways across diverse cancer types. F–H) Boxplots depicting the correlation of immune cell infiltration with FSS and TMB (ns, not significant; *p < 0.05, **p < 0.01, ***p < 0.001, ****p < 0.0001).

Given the substantial correlation between senescent CAFs and suppressed TME, we proposed that an FSS representing the senescent level of the CAFs in the tumor could facilitate the prognostication of ICI effectiveness. To that end, we utilized 40 scRNA‐Seq datasets to formulate the FSS (**Figure** **3**A; and Table S1, Supporting Information). Among these pan‐cancer scRNA‐seq datasets, we conducted Spearman correlation analysis to examine the correlation between gene expression levels and senescence score in fibroblasts. Genes showing a positive correlation with senescence score (Spearman *R* > 0 and FDR < 0.05) were identified as *FS_x_*, while genes exhibiting differential upregulation in fibroblasts were labeled as *FS_y_*. By intersecting *FS_x_* and *FS_y_*, we derived *FS_n_* (Table S2, Supporting Information), representing up‐regulated fibroblasts‐specific genes positively linked with senescence for each dataset.^[^ ^20^ ^]^ For instance, *FS*_1_ encompassed genes obtained from the intersection of *FS_x_* and *FS_y_* in the first scRNA‐seq dataset. The geometric mean of Spearman *R* values was computed for each gene across *FS*_1_–*FS*_40_. Subsequently, 109 genes with a geometric mean of Spearman *R* > 0.25 were aggregated as FSS (Table S3, Supporting Information). We further explored the biological events included in FSS via pathway enrichment analysis of Gene Ontology (GO) and the Kyoto Encyclopedia of Genes and Genomes (KEGG). As is shown in Figure 3B,C, the pathways enriched primarily in cell cycle, genetic remodeling, cancer‐related, and ICI‐related processes, which is aligned with prior findings related to the enhanced adhesive traits of senescent fibroblasts.^[^ ^1^ ^]^

### 2.3 Exploration of the Correlation between FSS and Immune Suppression

To delve deeper into the correlation between FSS and TME, we calculated the FSS scores for each patient from TCGA datasets across 30 cancer types based on the 109 FSS genes via the GSVA R package. Generally, our analysis revealed that solid tumors like head and neck squamous cell carcinoma (HNSCC), breast cancer (BRCA), and bladder cancer (BLCA) displayed higher FSS scores, whereas testicular germ cell tumors (TCGT), pheochromocytoma and paraganglioma (PCPG), and kidney renal papillary cell carcinoma (KIRP) exhibited lower scores (Figure S2, Supporting Information). Then, the R package MCPcounter was used to assess the abundance of immune cells, while tumors exhibiting high FSS scores showed reduced infiltration of antitumor immune cells, including CD8^+^ T cells, NK cells, and macrophages (Figure 3D). Subsequently, we conducted a correlation analysis between FSS scores and HALLMARK gene sets to explore whether immunosuppressive pathways were enriched in tumors with high FSS scores. It was found that top‐ranked pathways, such as TGF‐β signaling, apoptosis, Notch signaling, and hypoxia pathway were upregulated in patients with high FSS scores (Figure 3E).

Additionally, a range of analyses were conducted comparing tumor mutation burden (TMB) and FSS. Patients were classified into 8 subgroups based on the median value of FSS scores and TMB among TCGA pan‐cancer datasets for comparison: high FSS (HF), low FSS (LF), high TMB (HT), low TMB (LT), high FSS – high TMB (HFHT), high FSS – low TMB (HFLT), low FSS – high TMB (LFHT), low FSS – low TMB (LFLT). High FSS indicates a poor ICI response while high TMB takes the opposite stance. As expected, decreased infiltration of cytotoxic lymphocytes was observed in the HF and LT subgroups (Figure 3F,G). In addition, the comprehensive examination demonstrated that LFHT displayed a substantial presence of cytotoxic lymphocytes, while HFLT demonstrated the least amount (Figure 3H). Thus, the combination of High FSS and Low TMB (HFLT) might result in a deficient TME lacking cytotoxic lymphocytes. On the other hand, the LFHT subgroup showed enrichment in abundant cytotoxic lymphocytes. However, the immunological characteristics of HFHT and LFLT seemed more contentious than those of HFLT and LFHT. This discrepancy stemmed from the presence of both immune‐deficient (HF or LT) and immune‐competent (LF or HT) factors in HFHT and LFLT. To summarize, the hierarchy of immune system potency against tumors, arranged from greatest to least, is as follows: LFHT > LFLT > HFHT > HFLT (Figure 3H). Consequently, patients with lower FSS tended to exhibit superior antitumor immunity compared to those with higher FSS. In conclusion, we established a fibroblast‐specific, senescence‐related transcriptomic signature (FSS) serving as a pan‐cancer prognosticator for protumorigenic cell signaling, tumor‐promoting immune cell dysregulation, and ICI response.

### 2.4 Prediction of ICI Response Based on FSS

**Figure 4:**
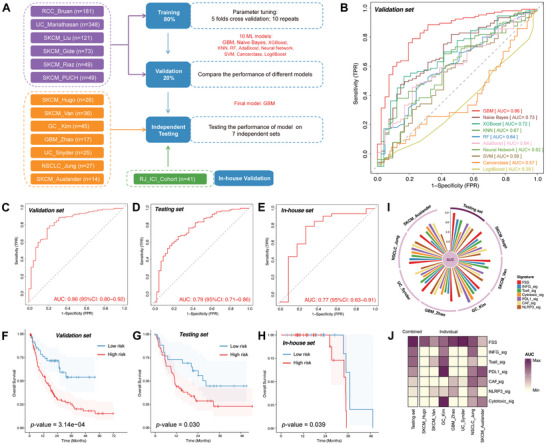
Prediction of ICI outcomes using FSS. A) Workflow of training, validating, and testing the FSS via 10 ML algorithms. B) Comparison of multiple ROC plots depicting the performance of final FSS in the validation set. ROC plot depicting the performance of the FSS model in validation C), testing D), and in‐house E) sets. Kaplan–Meier curves illustrating the survival analysis conducted between High‑risk and Low‑risk patients in validation F), testing G), and in‐house H) sets. I) Circos plot depicting the performance of other pan‑cancer signatures in the testing set. J) Heatmap comparing the predictive value of FSS and other pan‑cancer signatures.

To examine the potential of FSS in predicting the ICI response, we gathered 14 ICI cohorts containing complete sequencing data and immunotherapy clinical outcomes (patients were administered either anti‐PD(L)‐1, anti‐CTLA‐4, or a combination of anti‐PD(L)‐1 and anti‐CTLA‐4) (**Figure** **4**A). Initially, we merged 6 datasets (*n* = 821) from RCC_Bruan_Cohort (*n* = 181), UC_Mariathasan_Cohort (*n* = 348), SKCM_Liu_Cohort (*n* = 121), SKCM_Gide_Cohort (*n* = 73), SKCM_Riaz_Cohort (*n* = 49), and SKCM_PUCH_Cohort (*n* = 49). Next, we randomly divided the merged dataset into training (*n* = 657, 80%) and validation sets (*n* = 164, 20%) after removing the batch effect via the sva R package. Then, 10 MLs were executed using the caret R package and validated using 5 repetitions of 10‐fold CV. To enhance model accuracy, we underwent the optimization procedure 5 times using different random seeds. Following this, we calculated the values of the area under curve (AUC) for these models within the validation sets. Consequently, after performing these mathematical methods, we ultimately selected the gradient boosting machine (GBM) as the optimal model, which yielded the maximum AUC of 0.86 (Figure 4B,C). To further explore the strength of FSS, we applied the GBM method in the testing set (*n* = 190) and our in‐house set (*n* = 41), resulting in an AUC value of 0.79 and 0.77 (Figure 4D,E). Then, patients were categorized into high‐risk (predicted NR) and low‐risk (predicted R) groups for survival assessment. According to the Kaplan–Meier diagram, the low‐risk group exhibited favorable overall survival (OS) outcomes in validation, testing and in‐house sets (Figure 4F–H).

To assess the comprehensive value of FSS in predicting ICI response, we evaluated its efficacy against established pan‐cancer signatures (including INFG_sig,^[^ ^21^ ^]^ Tcell_sig,^[^ ^21^ ^]^ Cytotoxic_sig,^[^ ^22^ ^]^ PDL1_sig,^[^ ^23^ ^]^ CAF_sig,^[^ ^24^ ^]^ and NLRP3_sig).^[^ ^25^ ^]^ While most of these signatures demonstrated good predictive ability in individual datasets, FSS consistently demonstrated excellent performance among cohorts related to skin cutaneous melanoma (SKCM), glioblastoma multiforme (GBM), urothelial carcinoma (UC), gastric cancer (GC), and nonsmall cell lung cancer (NSCLC) (Figure 4I,J). Specifically, Cytotoxic_sig achieved AUC levels of 0.77 in Kim_GC_Cohort but dropped to 0.29–0.66 in other cohorts. In contrast, FSS consistently performed well in all enrolled cohorts (Table S4, Supporting Information). Our combined discoveries suggested that FSS acts as a robust predictive model across various cancers for responses to ICI treatment.

### 2.5 Survival Prognostication Based on FSS

**Figure 5:**
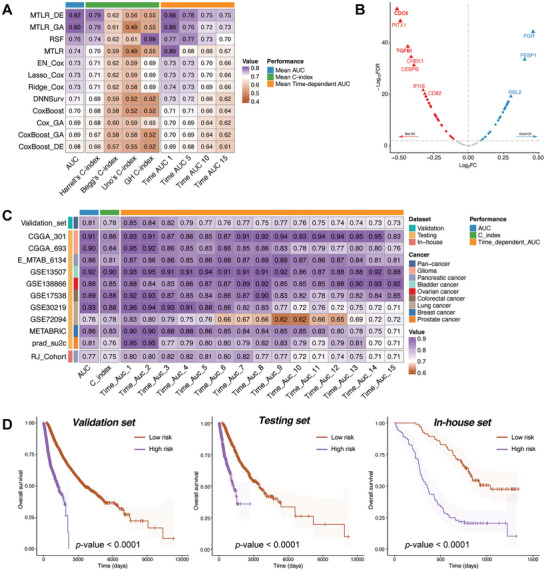
Prediction of prognosis based on FSS. A) Heatmap illustrating the C‐index of 15 ML‐generated models in the validation set. B) Volcano plot depicting the top 10 genes screened out from the MTLR_DE algorithm. C) Heatmap illustrating the C‐index of the final model in the validation, testing, and in‐house sets. D) Kaplan–Meier curves comparing OS between high‑risk and low‑risk patients in the validation, testing, and in‐house sets.

To optimize FSS for survival prognostication at the pan‐cancer level, the SurvBenchmark design was utilized to establish an FSS‐based survival model.^[^ ^18^ ^]^ 15 survival‐specific MLs were enrolled (Table S5, Supporting Information). The pan‐cancer patients from the TCGA were split into two groups: a training set comprising 80% (*n* = 7865) and a validation set comprising 20% (*n* = 1950). Utilizing the training set, a prognostic model based on FSS was developed, followed by the calculation of AUC, time‐dependent AUC, and C_index using the validation set. As shown in **Figure** **5**A, MTLR_DE demonstrated superior performance throughout the validation set and was identified as the optimal model. MTLR_DE represents a multitask logistic regression model with the ranking‐based method as a feature selection method. In this algorithm, the differential expression (DE) analysis between good and poor OS based on the median survival time across enrolled datasets was selected as a feature selection method, and 10 genes (CDC6, PITXQ, TGFBI, CHEK1, CEBPB, IFI16, CD82, PGR, PEBP1, and RBL2) were finally filtered out (Figure 5B). To validate the model based on the training set, we employed the MTLR algorithm via the MTLR R package, followed by the computation of individual risk scores for every patient. As is shown in Figure 5C, The outstanding performance of the final model was verified by the AUC values in validation, testing and in‐house sets. Additionally, individuals exhibiting elevated risk scores across all participant groups demonstrated poorer OS rates (Figure 5D; and Figure S3, Supporting Information), aligning consistently with most datasets within the TCGA pan‐cancer cohort (Figure S4, Supporting Information). A nomogram was further created by combining the FSS‐associated risk score with the American Joint Committee on Cancer (AJCC) stage to enhance the predictive accuracy of the aforementioned risk score in pan‐cancer datasets (Figure S5A, Supporting Information). As shown in Figure S5B (Supporting Information), the calibration curves for OS within the initial 5‐year period postcancer diagnosis demonstrated a strong correlation between predicted survival probability and observed survival rates, underscoring the reliability of this nomogram for prognosticating survival outcomes. Based on these findings, we have determined that the FSS‐based prognostic model could emphasize its capability as a predictive instrument in pan‐cancer datasets.

### 2.6 CRISPR/Cas9 Screening Identified Potential Targets by FSS

**Figure 6:**
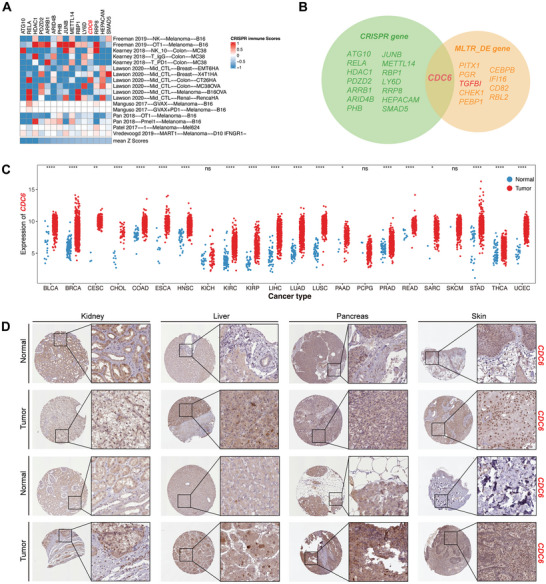
Exploration of potential targets from FSS via CRISPR/Cas9 datasets. A) Heatmap illustrating the z‐scores of 15 FSS genes in the 10% top‑ranked genes. B) Veen plot showing the intersection of FSS genes ranked in CRISPR/Cas9 datasets and genes screened out via the MTLR_DE algorithm. C) The mRNA expression of CDC6 across TCGA pan‐cancer datasets. D) The representative image of IHC of CDC6 in tumor samples of the kidney, liver, pancreas, and skin (ns, not significant; *p < 0.05, **p < 0.01, ***p < 0.001, ****p < 0.0001).

Identifying potential targets for ICI response is crucial for advancing the clinical application of FSS. To achieve this point, we split 17 CRISPR/Cas9 cohorts from 7 CRISPR/Cas9 research, categorizing knockout genes based on cell line types and treatment conditions while integrating ICI response information. The enrolled 22505 CRISPR genes underwent z‐score ordering, defining the top‐ranked ones as immune‐resistant genes (Figure S6A, Supporting Information). Essentially, top‐ranked genes may enhance antitumor immunity postknockout, whereas lower‐ranked ones might impede it. Comparisons were then made between the percentage of top‐ranked genes in FSS and those in established immune‐resistant signatures (including TcellExc_sig,^[^ ^20^ ^]^ ImmuneCells_sig,^[^ ^24^ ^]^ IMS_sig,^[^ ^26^ ^]^ CAF_sig,^[^ ^24^ ^]^ and CRMA_sig).^[^ ^27^ ^]^ Figure S6B (Supporting Information) illustrated that FSS contained the highest proportion of top‐ranked genes compared to other signatures. Intriguingly, the top 10% of genes (*n* = 15) were disproportionately represented in FSS (Fisher's exact test, *p* < 0.01). After undergoing additional validation in various independent CRISPR/Cas9 datasets, these genes surfaced as promising candidates for immunotherapeutic targeting (**Figure** **6**A). To further explore the targets for both predicting ICI response and survival, we analyzed the 15 genes chosen from CRISPR/Cas9 datasets and the 10 genes included in the FSS‐based prognostic model (Figure 5B). Eventually, CDC6 was intersected as the hub gene (Figure 6B). As illustrated in Figure 6C, in 19 out of 22 (86%) pan‐cancer cohorts, the mRNA level of CDC6 significantly increased in tumor specimens in contrast to those in normal tissues, and this elevated CDC6 expression was associated with unfavorable OS (Figure S7, Supporting Information). Analysis of the Human Protein Atlas (HPA) datasets through IHC also indicated increased protein expression levels of CDC6 in tumor samples of the kidney, liver, pancreas, and skin (Figure 6D).

### 2.7 Downregulation of CDC6 in PC Cells Potentiates the Response to Immunotherapy

**Figure 7:**
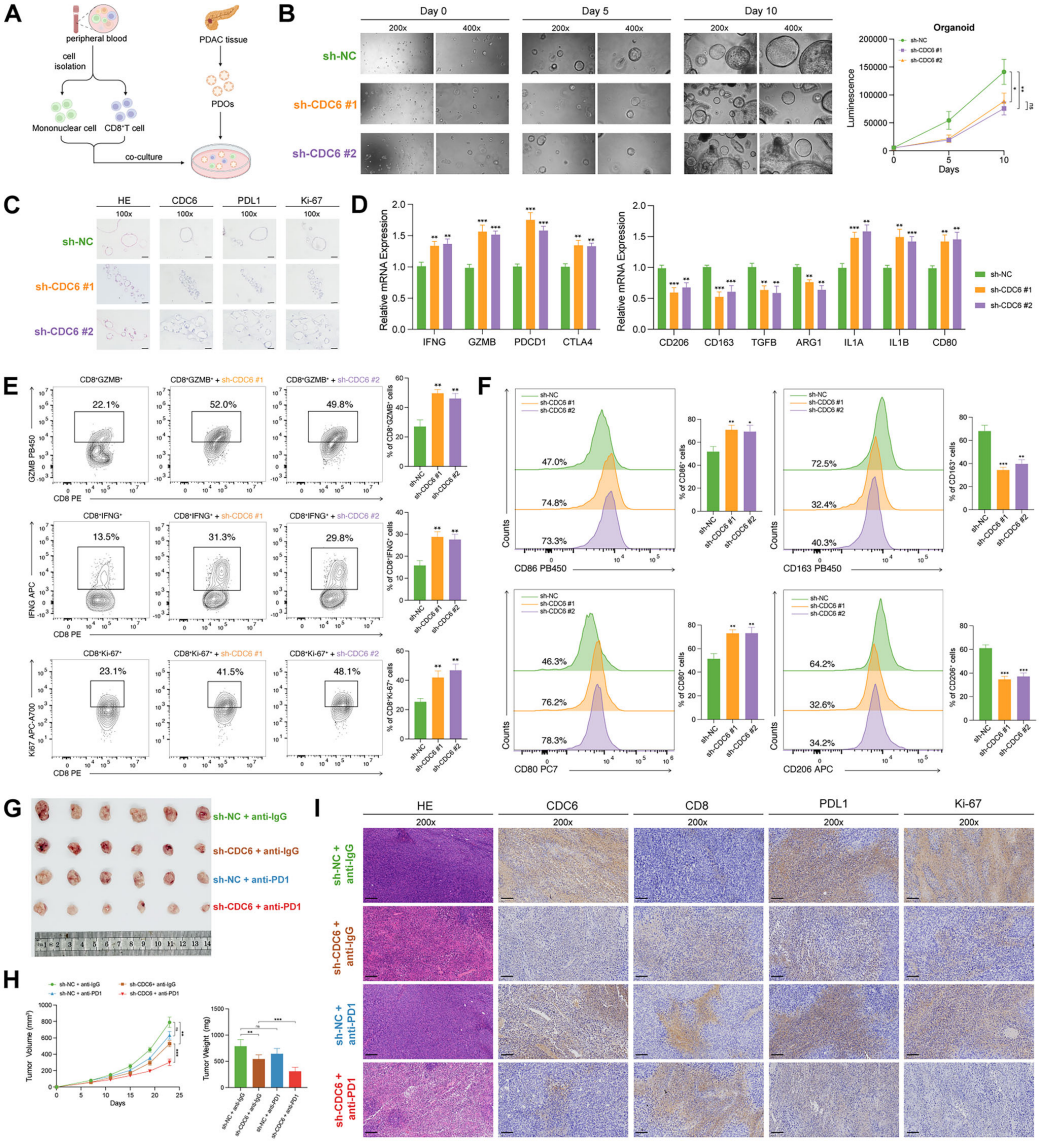
CDC6 deficiency in PC enhanced the antitumor response. A) The workflow illustrating the cocultivation of immune cells with PC organoids. B) Observation under the microscope (Left panel) and CTG assay (Right panel) revealed that the downregulation of CDC6 significantly decreased the growth rate of PC organoids. C) IHC staining depicting the expression of CDC6, PDL1, and Ki‐67 in the sh‐NC and sh‐CDC6 groups (scale bar: 100 µm). D) qRT‐PCR analysis unveiled the expression profiles of markers in sh‐NC and sh‐CDC6 PC organoids. E) Representative plots and percentages of CD8^+^GZMB^+^, CD8^+^IFNG^+^, and CD8^+^Ki‐67^+^ cells from sh‐NC and sh‐CDC6 groups. F) Representative histograms and percentages of CD86^+^, CD80^+^, CD163^+^, and CD206^+^ cells from sh‐NC and sh‐CDC6 groups. G) Images representing subcutaneous PC tumors from mice administered with sh‐NC+anti‐IgG, sh‐CDC6+anti‐IgG, sh‐NC+anti‐PD1, and sh‐CDC6+anti‐PD1. H) Tumor growth rate (Left panel) and weight (Right panel) in response to treatment with sh‐NC+anti‐IgG, sh‐CDC6+anti‐IgG, sh‐NC+anti‐PD1, and sh‐CDC6+anti‐PD1. I) IHC staining depicting the expression levels of CDC6, CD8, PDL1, and Ki‐67 across sh‐NC+anti‐IgG, sh‐CDC6+anti‐IgG, sh‐NC+anti‐PD1, and sh‐CDC6+anti‐PD1 groups (scale bar: 100 µm). (ns, not significant; *p < 0.05, **p < 0.01, ***p < 0.001, ****p < 0.0001).

To elucidate the role of CDC6 in antitumor immunity, we established a coculture system comprising CD8^+^ T cells and CD14^+^ monocyte‐derived macrophages with PC organoids (**Figure** **7**A). Following CDC6 knockdown in PC organoids, we confirmed significant reductions in both CDC6 mRNA and protein expression (Figure S8A,B, Supporting Information). Notably, the expression of p16, a canonical senescence marker,^[^ ^28^ ^]^ was concomitantly decreased, suggesting that CDC6 silencing might attenuate fibroblast senescence within the TME (Figure S8B, Supporting Information). Morphological assessment and CTG analyses demonstrated significantly impaired growth kinetics in sh‐CDC6 PC organoids (Figure 7B). Furthermore, IHC analysis revealed markedly reduced expression of PD‐L1 and Ki‐67 in sh‐CDC6 organoids compared to controls (Figure 7C).

qRT‐PCR analysis of the coculture system revealed significant upregulation of ICI‐related genes in cells interacting with sh‐CDC6 organoids. Concurrently, we observed diminished expression of M2 macrophage‐associated markers alongside elevated M1 macrophage markers (Figure 7D), indicating a shift from pro‐tumoral to anti‐tumoral macrophage polarization. Flow cytometric analyses further corroborated these findings, demonstrating enhanced CD8^+^ T cell activation and proliferation in sh‐CDC6 organoids (Figure 7E), accompanied by preferential M1 macrophage polarization compared to the sh‐NC group (Figure 7F). To validate these observations in vivo, we established KPC subcutaneous xenografts in C57BL/6 mice and administered anti‐IgG or anti‐PD1 antibodies intraperitoneally. Notably, CDC6‐deficient tumors exhibited markedly enhanced sensitivity to anti‐PD1 therapy, as evidenced by superior tumor regression (Figure 7G,H). IHC analysis of the tumor specimens revealed increased immune cell infiltration coupled with reduced proliferative indices in CDC6‐deficient tumors (Figure 7I). Collectively, these findings underscore the critical role of CDC6 in modulating the immune landscape within the TME and suggest that its suppression may potentiate immunotherapeutic efficacy in pancreatic cancer.

### 2.8 Tumor Cell‐Derived CDC6 Drives Fibroblast Senescence via TGF‐β1 Secretion and Oxidative Stress

**Figure 8:**
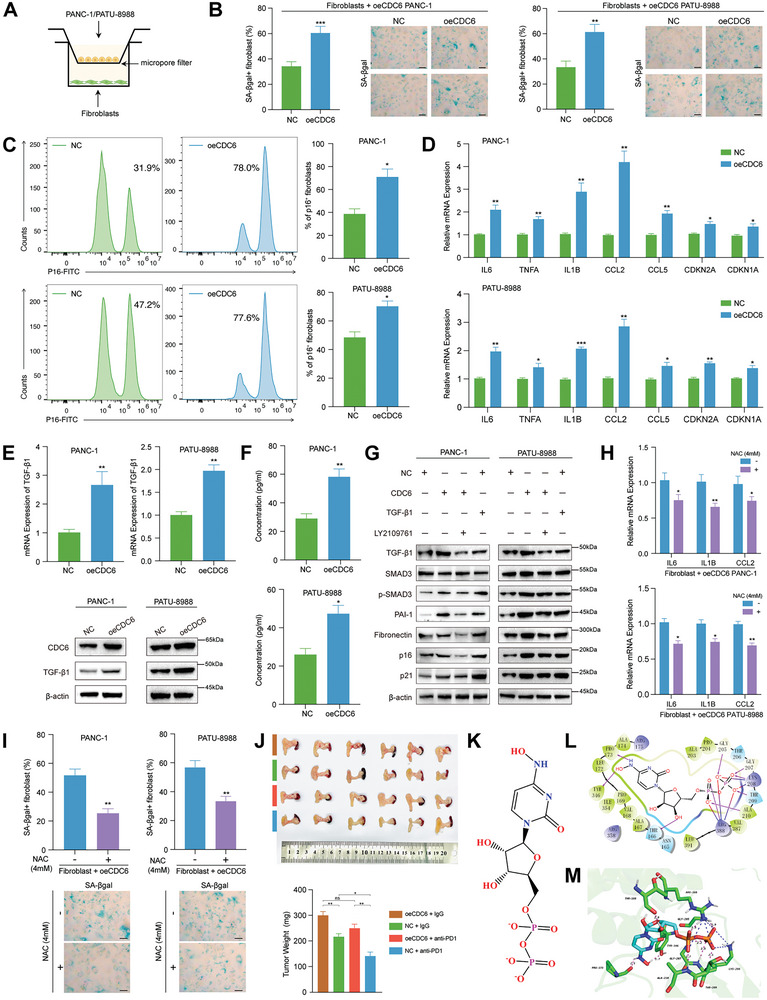
CDC6 promotes fibroblast senescence through TGF‐β1 secretion and oxidative stress. A) Schematic of the tumor cell‐fibroblast coculture system with transwells. B) Percentage of SA‐βgal^+^ fibroblasts following coculture with oeCDC6 PANC‐1 or PATU‐8988 cells (scale bar: 100 µm). C) Representative plots and percentages of p16^+^ fibroblasts in coculture with oeCDC6 PANC‐1 or PATU‐8988 cells. D) qRT‐PCR analysis showing the expression profiles of SASP markers in the coculture system with oeCDC6 PANC‐1 or PATU‐8988 cells. E) mRNA and protein expression levels of TGF‐β1 in the coculture system with oeCDC6 PANC‐1 or PATU‐8988 cells. F) ELISA analysis showing the concentration of TGF‐β1 in the coculture system with oeCDC6 PANC‐1 or PATU‐8988 cells. G) Protein expression of markers in the TGF‐β signaling pathway. H) mRNA expression of IL6, IL1B, and CCL2 in the coculture system. I) The senescent phenotype of fibroblasts cocultured with PANC‐1 or PATU‐8988 cells, with or without 4 mm NAC, as indicated by the percentage of SA‐βgal^+^ cells (scale bar: 100 µm). J) Tumor growth weight in response to treatments with oeCDC6+IgG, NC+IgG, oeCDC6+anti‐PD1, and NC+anti‐PD1 (*n* = 6). K) Chemical structure of NHC‐diphosphate (triammonium). In silico docking of NHC‐diphosphate (triammonium) into the active site of the human CDC6 protein in 2D L) and 3D M).

To investigate the functional relationship between CDC6 and fibroblast senescence, we established a tumor cell‐fibroblast coculture system (**Figure** **8**A). CDC6 overexpression in PC cells markedly induced senescence in cocultured fibroblasts, as demonstrated by enhanced SA‐β‐galactosidase activity (Figure 8B), increased p16^+^ fibroblast population (Figure 8C), and elevated expression of senescence‐associated secretory phenotype (SASP) factors (Figure 8D).

Given the well‐established role of TGF‐β1 as a canonical regulator of cellular senescence,^[^ ^29^ ^]^ coupled with our findings that TGF‐β signaling ranked first among FSS‐related pathways (Figure 3E) and emerged as a top candidate in the MTLR_DE model (Figure 5B), we investigated its potential contribution to CDC6‐mediated fibroblast senescence. Results confirmed that CDC6‐overexpressing tumor cells exhibited significantly elevated TGF‐β1 levels both intracellularly and in the coculture medium (Figure 8E), which was further validated by ELISA (Figure 8F). The cocultured fibroblasts displayed activation of the TGF‐β‐SMAD2/3 signaling cascade, triggering fibrosis and senescence programs (Figure 8G). Notably, pharmacological inhibition of TGF‐β signaling with LY2109761 abolished CDC6‐induced fibroblast senescence (Figure 8G), indicating that TGF‐β1 is a critical mediator in this process.

Since CDC6 has been implicated in stress‐induced senescence,^[^ ^30^ ^]^ we next examined the contribution of oxidative stress. N‐acetylcysteine (NAC) has been well‐documented as a potent antioxidant capable of suppressing oxidative stress responses.^[^ ^31^ ^]^ To elucidate the potential role of oxidative stress in this process, we supplemented the coculture system with NAC and investigated its effects on CDC6‐mediated cellular senescence. Results revealed that NAC treatment significantly reduced fibroblast senescence in cocultures with PC cells, as indicated by decreased SASP factor expression (Figure 8H) and a lower proportion of SA‐βgal^+^ cells (Figure 8I).

Finally, in an orthotopic mouse model of PC, high CDC6 expression was associated with more severe fibroblast senescence and a diminished response to ICI therapy (Figure 8J). These findings suggest that CDC6 modulates ICI response by regulating oxidative stress in CDC6/TGF‐β1‐induced fibroblast senescence.

### 2.9 High‐Throughput Virtual Screening (HTVS) of CDC6 Inhibitor

Currently, no commercially available inhibitors targeting CDC6 exist. To address this, we performed a structure‐based HTVS of 23100 compounds to identify potential CDC6 inhibitors. Sequential standard precision (SP) and extra precision (XP) docking analyses were conducted, leading to the selection of top‐ranked compounds. Among these, β‐D‐N(4)‐hydroxycytidine (NHC)‐diphosphate (triammonium) demonstrated the strongest inhibitory effect on CDC6 protein expression (Figure 8K; and Table S6, Supporting Information). NHC‐diphosphate (triammonium) interacts with the CDC6 through 9 hydrogen bonds and 5 salt bridges. Multiple phosphate oxygen atoms act as hydrogen bond acceptors, forming 6 hydrogen bonds with GLY205, GLY207, LYS208, THR209, ALA210, and ARG388, with bond distances of 1.9, 2.2, 2.5, 2.1, 1.7, and 1.5 Å, respectively. 2 hydroxyl groups serve as hydrogen bond donors, forming 2 hydrogen bonds with THR166 and TYR346, with distances of 1.9 and 1.7 Å, respectively. Additionally, an amino hydrogen atom acts as a hydrogen bond donor, forming one hydrogen bond with PRO173 at a distance of 2.1 Å. Moreover, NHC‐diphosphate (triammonium) forms two salt bridges with LYS208 and three salt bridges with ARG388 (Figure 8L,M).

## 3 Discussion

Assessing the individual prognosis and forecasting ICI response for patients play pivotal roles in shaping personalized treatment approaches within precision oncology. Presently, a growing cohort of researchers has shifted their attention to the immunosuppressive impact of CAFs, accomplished through their engagement with elements of the TME, notably immune cells.^[^ ^32^, ^33^ ^]^ An instance demonstrated how CAFs may impede the penetration of immune cells, including CD8^+^ T lymphocytes, into tumor locations through the secretion of diverse chemokines.^[^ ^34^ ^]^ Additionally, the ratios of immune‐suppressing entities, such as M2 macrophages, regulatory T cells (Tregs), and myeloid‐derived suppressor cells (MDSCs), influenced CAFs, have demonstrated notable escalation within the TME, consequently fostering immune evasion mechanisms within the tumor milieu.^[^ ^35^ ^]^ Moreover, certain cytokines released by immune cells upon activation, like interleukin (IL)‐1β, have the capacity to prompt the conversion of regular fibroblasts into proinflammatory CAFs. This process also enhances the attraction of inhibitory immune cells, leading to immune suppression within the TME.^[^ ^36^ ^]^ The functional attributes of cells undergo substantial changes due to cellular senescence, which has recently been associated with the fundamental features of cancer, serving as a vital element in solid tumor development.^[^ ^7^ ^]^ In pursuit of this objective, senescent tumor cells attract immune cells that suppress the immune system through the secretion of various molecular substances.^[^ ^37^ ^]^ Fascinatingly, it has been noted that senescence within tumor cells also triggers CD8^+^ T cells by releasing alarmins, initiating interferon signaling and displaying self‐peptides associated with senescence. This process ultimately encourages the eradication of tumor cells.^[^ ^37^ ^]^ In addition to tumor cells, the cellular milieu within tumors, encompassing fibroblasts, immune cells, and endothelial cells, experiences changes associated with senescence, significantly impacting tumor advancement: senescent CAFs promote tumor expansion and infiltration by releasing cytokines and extracellular vesicles.^[^ ^15^ ^]^ Therefore, the senescent process of CAFs could potentially become a promising focus for predicting survival outcomes and assessing the efficacy of ICI therapy in cancer treatment, and may also represent a viable therapeutic strategy.

To explore the predictive and prognostic potential of senescence in CAFs, our initial step involved analyzing 40 scRNAseq datasets. We assessed the level of cellular senescence within distinct cell populations in solid tumors spanning 17 cancer types. To accomplish this objective, we utilized a well‐established gene signature associated with fibroblast senescence—unlike other gene signatures related to senescence, this one comprehensively encompasses transcriptomic alterations linked to senescence across various contexts. Our transcriptomic investigations unveiled that within the TME of malignant tumors, CAFs showcased the most pronounced levels of senescence compared to other cellular populations. Given the disorganized nature of tumor microvessels and their aberrant functional traits, such as irregular immune cell interactions, cellular senescence could significantly exacerbate these pathological characteristics observed in CAFs. Through the cell–cell communication analysis, it is demonstrated that HS‐CAF consistently engaged in interactions with diverse cellular populations, often comprising immune cells, in contrast to CAFs exhibiting diminished levels of cellular senescence. Notably, this interaction primarily entails immune cells possessing tumor‐promoting characteristics, such as myeloid cells. Accordingly, our findings proposed that CAFs are susceptible to experiencing cellular senescence, leading to disrupted immune monitoring in solid tumors.

From these discoveries, an innovative transcriptomic signature unique to CAFs was formulated, comprising 109 genes, termed FSS. Functionally, these genetic components predominantly govern the cell cycle, DNA remodeling and tumorigenesis. In line with earlier findings, it has been documented that senescent CAFs promote epithelial cell growth and tumorigenesis.^[^ ^15^ ^]^

Next, we analyzed how FSS correlates with the frequency of particular biological characteristics, the emergence of diverse immune cell reactions, and survival. Our investigation revealed a positive correlation between FSS and various tumorigenic pathways in TCGA pan‐cancer datasets. Within these pathways that promote tumorigenesis, both TGF‐β and the Notch signaling pathways have not only demonstrated links to the proliferation of tumors but also serve as significant controllers of fibroblast senescence.^[^ ^38^ ^]^ Moreover, FSS was associated with irregular infiltration of CD8^+^ T cells within tumors, a factor deemed crucial for the effectiveness of ICI therapy.^[^ ^39^ ^]^ Regarding the immunomodulatory capacity of senescent CAFs, our FSS derived from CAFs could potentially forecast the efficacy of ICI treatments as well. Here, FSS forecasts the reaction to ICI therapy among lung cancer, stomach cancer, urothelial carcinoma, kidney cell carcinoma, basal cell carcinoma, and PC. Within this framework, FSS exhibited high AUC values across various datasets, surpassing the efficacy of prior pan‐cancer predictive signatures for ICI treatment effectiveness, which were limited to achieving satisfactory results in only a handful of datasets. To validate the prognosis prognostication of FSS, the MTLR_DE algorithm in SurvBenchmark design was implemented on pan‐cancer datasets from TCGA. This analysis uncovered a correlation between elevated risk scores and poorer OS outcomes. Collaboratively, we showcased the outstanding performance of FSS in forecasting ICI outcomes and predicting survival rates among patients with various types of cancer.

Due to the remarkable performance exhibited by FSS in forecasting the outcomes of immunotherapy, an imperative exists to pinpoint potential targets within FSS. Therefore, we employed CRISPR/Cas9 datasets and arranged genes according to their logFC of small guide RNA (sgRNA) readings under conditions of immune competence or deficiency. After correlating the genes with the highest ranking in CRISPR/Cas9 datasets and those prioritized in the prognostic model related to FSS, CDC6 emerged as the hub gene for further analyses. Cell cycle regulator 6 (CDC6), located on chromosome 17q21.3, serves as a crucial licensing agent for DNA replication in both the G1 and S phases of eukaryotic cell division.^[^ ^40^ ^]^ Numerous prior investigations have indicated that irregular CDC6 contributes to oncogenic processes across a range of malignancies, potentially serving as a diagnostic and prognostic indicator for associated tumors.^[^ ^41^, ^42^, ^43^ ^]^

In our study, we highlight the pivotal role of tumor cell‐derived CDC6 in driving fibroblast senescence via TGF‐β1 secretion and oxidative stress. Coculture experiments revealed that CDC6 overexpression in PC cells induces fibroblast senescence, characterized by increased SA‐βgal activity, p16 expression, and a pro‐inflammatory SASP profile. TGF‐β1 was identified as a key mediator,^[^ ^29^ ^]^ with its upregulation in tumor cells activating the TGF‐β‐SMAD2/3 pathway in fibroblasts. Oxidative stress further amplified senescence, as NAC treatment reduced senescence markers and SASP expression. These findings indicate that CDC6 induces fibroblast senescence through a dual mechanism involving TGF‐β signaling and oxidative stress. In vivo, high CDC6 expression correlated with increased fibroblast senescence and diminished response to ICI, highlighting the clinical relevance of CDC6‐induced stromal remodeling. Finally, HTVS identified NHC‐diphosphate (triammonium) as a potential CDC6 inhibitor, with strong binding predicted through extensive hydrogen bonds and salt bridges. NHC is a modified cytidine derivative, where the N(4)‐hydroxy modification alters the cytosine base. A recent study highlights that UDP‐driven signaling pathways, mediated by cytidine deaminase (CDA) and P2Y_6_ receptor, are critical in tumor‐associated macrophage (TAM) immunosuppression, contributing to ICI resistance in PC.^[^ ^44^ ^]^ NHC‐diphosphate (triammonium), as a nucleotide analog, could potentially reprogram the TME to affect the ICI response. These findings provide a foundation for developing targeted therapies to disrupt CDC6‐mediated tumor‐stromal interactions.

Crucially, our research faces several constraints. In this study, while the role of CAFs in the TME has been thoroughly explored, other cell types, such as immune cells, stromal cells, and even tumor cells themselves, which are also critical for predicting ICI response, have not been sufficiently considered. Future research should aim to integrate these additional factors, particularly the roles of immune and stromal cells in the TME, to provide a more comprehensive assessment of tumor response to ICI. Additionally, while the selection of the CDC6 inhibitor was supported by both computational data and its mechanistic relevance as a nucleotide analog, experimental validation will be required to confirm its inhibitory effects on CDC6 and its potential to enhance ICI efficacy in our future work.

## 4 Conclusion

In conclusion, our study provides new insights into the molecular mechanisms underlying tumor senescence, emphasizing the clinical significance of the FSS in predicting patient survival and response to ICI therapy. Among the identified targets, CDC6 stands out as a critical regulator of fibroblast senescence, driven by TGF‐β signaling and oxidative stress. These findings underscore the potential of targeting CDC6 and its downstream effects to improve therapeutic outcomes in precision oncology.

## 5 Experimental Section

### Patients

This research followed the Declaration of Helsinki and was approved by the Ethics Committee of Ruijin Hospital, Shanghai Jiao Tong University School of Medicine, Shanghai, China. Consent was obtained from all participants whose samples were collected for sequencing, as the study was observational and noninterventional. A total of 41 patients who received immunotherapy were included, named “RJ_ICI_Cohort.” All enrolled eligible patients meet the following criteria: i) Histo‐pathologically confirmed PC; ii) no other malignant history; iii) follow‐up was completed within the scheduled time frame. iv) those who were immunotherapy treated received neoadjuvant PD‐1 therapy.

### Sample Collection

Primary PC tumor tissues were resected surgically and placed in sterile vials. Samples experienced cold ischemia for under 30 min, with freezing in liquid nitrogen averaging 20 min. In addition to snap‐freezing and storage at −80 °C for further analysis, formalin fixation was used for H&E staining and histological review.

### Treatment and Follow‐Up

The decision to administer anti‐PD‐1 instead of upfront surgery was made by multidisciplinary tumor boards at participating centers through rigorous discussion. Given that current NCCN guidelines (Version 3.2024) do not include anti‐PD‐1 in the standard first‐line treatment algorithm for PC, this therapeutic strategy was implemented within the context of clinical investigation, following careful evaluation of patients’ clinicopathological characteristics, and molecular profiles.

### Cell Culture

KPC1199, PANC‐1, and PATU‐8988 cells were cultured by supplementing DMEM with 10% FBS and 1% Penicillin–Streptomycin Solution (P/S) at 37 °C in a 5% CO_2_ humidified incubator. The STR cell authentication method was employed to identify all cell lines, and regular testing for mycoplasma contamination was conducted utilizing the PCR Mycoplasma Detection Kit (G238, Applied Biological Materials). To ensure stable transfections, the suitable lentivirus was introduced into the supernatant, and within 6–8 h, the medium underwent alteration. Upon confirming expression, cells underwent puromycin treatment at a concentration of 2 µg mL^−1^ to distinguish those harboring the resistance gene, indicative of established transfection success.

### Coculture of Cancer Cells and Fibroblasts

A tumor cell‐fibroblast coculture system was developed using Transwell inserts (Corning). Fibroblast cells were seeded on collagen‐coated wells in the lower chamber, while cancer cells were plated into the inserts at a density of 2.2 × 10^4^ cells cm^−2^ using a serum‐free medium. After 2 days of coculture, allowing interaction via soluble factors, fibroblast cells were further analyzed. For flow cytometry analysis, duplicate samples were included for consistency.

### Construction of Patient‐Derived PC Organoids

PC organoids were cultured from specimens taken from patients diagnosed with PC who underwent surgical procedures at Ruijin Hospital. Tumor specimens obtained from patients were immediately sectioned into small fragments in pre‐chilled RPMI‐1640 medium and digested at 37 °C for 20 min into single‐cell suspension. Organoids were seeded into 24‐well plates in a mixture of Matrigel (Corning) and RPMI‐1640. Upon the gelation process's completion, 800 µL organoid culture medium was introduced into every well. Subsequently, the plates were relocated to incubators with a humidity of 37 °C/5% CO_2_. Every 4 days, the medium underwent alteration, while the organoids experienced passaging intervals ranging from 1 to 2 weeks.

### RNA Sequencing Analysis

RNA libraries were prepared using the Kapa RNA HyperPrep Kit with RiboErase. A total of 500 ng RNA was fragmented to 200–300 bp at 94 °C for 6 min. The fragmented RNA was reverse transcribed into first‐strand cDNA using random primers, followed by second‐strand synthesis to form double‐stranded cDNA. RNA‐Seq libraries were sequenced on the Illumina NovaSeq 6000 platform (Illumina). For RNA‐seq data analysis, reads were mapped to the human reference genome GRCh38, with both the reference and annotation files sourced from the GENECODE database (https://www.gencodegenes.org/human/). STAR^[^ ^45^ ^]^ was used for alignment, and RSEM^[^ ^46^ ^]^ was employed to generate transcripts per kilobase of exon per million mapped reads (TPM) and z‐score normalization was implemented.

### Pan‐Cancer scRNA‐seq and Bulk RNA‐seq Cohorts

A collection of 40 pan‐cancer scRNA‐seq cohorts, comprising 406 individuals and 881 332 cells, was gathered to construct a distinctive signature specific to fibroblast senescence. Seurat,^[^ ^47^ ^]^ a standard R package, was utilized to process these extensive scRNA‐seq datasets encompassing 17 cancer types. Raw data extraction was performed from Gene Expression Omnibus (GEO), Array Express and The European Genome‐phenome Archive (EGA). To ensure the integrity of the research, cells exhibiting gene expression of fewer than 500 genes along with those displaying mitochondrial gene expression exceeding 5% were removed from the analysis. Principal component analysis (PCA) was utilized on the dataset for dimensionality reduction, with the elbowplot function employed to determine the optimal number of significant principal components. These components were then employed for cell clustering and generating t‐SNE for dimensionality reduction. Cells were annotated with known markers or downloaded from their original datasets. Additionally, the FindAllMarkers function was utilized to identify distinct genes within each cluster employing thresholds of default values (logFC > 0.25 and min.pct > 0.1). See detailed information in Table S1 (Supporting Information). Transcriptomic data from 30 TCGA cohorts, comprising 9815 patients with complete clinicalpathological data, was obtained from the UCSC Xena portal (https://xenabrowser.net), after normalizing batch effects. Acquiring the TMB data of enrolled TCGA cohorts was facilitated via the cBioPortal (https://www.cbioportal.org). Clinicalpathological and bulk RNA‐seq data from another 10 cohorts, totaling 4434 patients, were gathered as testing sets to assess the prognostic model related to FSS. In addition, another in‐house RNA‐seq dataset named “RJ_Cohort” (*n* = 185) was utilized for external validation.^[^ ^48^ ^]^ Raw transcriptome data for these cohorts were sourced from their correlated literature. The normalization of the expression matrix was conducted using z‐score across all cohorts. See detailed information in Table S7 (Supporting Information).

### Bulk ICI RNA‐seq Cohorts

To validate the predictive significance of FSS in ICI response, transcriptomic data and clinical details from pretreatment 1052 samples of 14 ICI RNA‐Seq cohorts, including 1 Renal Cell Carcinoma (RCC) cohort (RCC_Bruan_Cohort),^[^ ^49^ ^]^ 7 SKCM cohorts (SKCM_Liu_Cohort,^[^ ^50^ ^]^ SKCM_Gide_Cohort,^[^ ^51^ ^]^ SKCM_Riaz_Cohort,^[^ ^52^ ^]^ SKCM_PUCH_Cohort,^[^ ^26^ ^]^ SKCM_Hugo_Cohort,^[^ ^53^ ^]^ SKCM_Van_Cohort,^[^ ^54^ ^]^ and SKCM_Auslander_Cohort),^[^ ^55^ ^]^ 2 UC cohorts (UC_Mariathasan_Cohort^[^ ^56^ ^]^ and UC_Snyder_Cohort),^[^ ^57^ ^]^ 1 GC cohort (GC_Kim_Cohort),^[^ ^58^ ^]^ 1 GBM cohort (GBM_Zhao_Cohort),^[^ ^59^ ^]^ 1 NSCLC cohort (NSCLC_Jung_Cohort),^[^ ^60^ ^]^ and 1 PC in‐house set (RJ_ICI_Cohort) were systematically gathered. See detailed information in Table S8 (Supporting Information).

### CRISPR/Cas9 Screening Datasets

Our research included 17 rearranged datasets extracted from 7 CRISPR/Cas9 libraries focusing on the effects of antitumor immunity after CRISPR/Cas9 knockout screens. This research was carried out by Freeman,^[^ ^61^ ^]^ Kearney,^[^ ^62^ ^]^ Manguso,^[^ ^63^ ^]^ Pan,^[^ ^64^ ^]^ Patel,^[^ ^65^ ^]^ Vredevoogd,^[^ ^66^ ^]^ and Lawson,^[^ ^67^ ^]^ and covered multiple cancer cell lines. In this study, the initial 6 research summarized by Fu^[^ ^68^ ^]^ and an extra CRISPR/Cas9 research from Lawson^[^ ^67^ ^]^ were enrolled. Finally this research was restructured into 17 datasets based on their conditions. The extensive CRISPR/Cas9 analysis aimed to pinpoint genes more likely to influence antitumor immunity and impact ICI response across different groups. logFC in sgRNA readings were calculated between CTLs versus non‐CTLs or immune‐competent versus immune‐deficient subsets, providing a metric to evaluate cancer fitness postgene knockout. Z‐score normalization was implemented on logFC of enrolled CRISPR/Cas9 datasets to remove batch effects. After gene knockout, a more robust immune response is apparent, indicated by decreased z‐scores. Genes exhibiting the highest z‐scores are pinpointed as showcasing resilience against processes linked to the immune system. See detailed information in Table S9 (Supporting Information).

### Pathway, Antitumor Immunity, and Cell–Cell Communication Analysis

The GSVA^[^ ^69^ ^]^ R package was employed to conduct GSVA. GSVA is a nonparametric, unsupervised method used to estimate the variation of pathway or gene set activity over a sample population in an expression dataset. It transforms gene expression data from individual genes into pathway‐level information, allowing researchers to assess the activity of predefined sets of genes across samples. The cell senescence‐induced genes were extracted from the CellAge^[^ ^70^ ^]^ dataset (https://genomics.senescence.info/cells). GO and KEGG analyses were carried out using the R package clusterProfiler.^[^ ^71^ ^]^ R package MCPcounter^[^ ^72^ ^]^ was employed to quantification of the absolute abundance of immune and stromal cell populations from transcriptomic data. CellChat^[^ ^73^ ^]^ R package was employed for assessing intercellular communication by utilizing the standardized gene expression data. Interaction combinations were kept involving HS‐CAF, LS‐CAF, and various cell categories where the statistical significance (*p* < 0.01) was observed.

### Establishment of a Predictive Model for ICI Response—Cohorts

To access the performance of FSS in predicting ICI response, a meta‐cohort (*n* = 821) was formed by merging the 6 largest ICI cohorts (RCC_Bruan_Cohort, UC_Mariathasan_Cohort, SKCM_Liu_Cohort, SKCM_Gide_Cohort, SKCM_Riaz_Cohort, and SKCM_PUCH_Cohort) after removing the batch effect based on sva R package. Then, patients in meta‐cohort were categorized into a training set (*n* = 657) and a validation set (*n* = 164) at a ratio of 8:2, while the remaining 7 ICI cohorts (SKCM_Hugo_Cohort, SKCM_Van_Cohort, GC_Kim_Cohort, GBM_Zhao_Cohort, UC_Snyder_Cohort, NSCLC_Jung_Cohort, and SKCM_Auslander_Cohort) were designated as testing sets (*n* = 190). In addition, RJ_ICI_Cohort (*n* = 41) was also implemented to validate as an in‐house set (Table S8, Supporting Information).

### Establishment of a Predictive Model for ICI Response—Model Training, Validation, and Testing

Based on the FSS, 10 ML algorithms, including GBM, Naïve Bayes (NB), eXtreme Gradient Boosting (XGBoost), *k*‐nearest neighbors (KNN), Random Forest (RF), Adaptive Boosting (AdaBoost), Neural Network, Support Vector Machine (SVM), Cancerclass, and Logistic Boosting (LogiBoost), were employed for model training. fivefold cross‐validation (CV) with 10 iterations per resampling for robustness was applied. The caret^[^ ^74^ ^]^ R package facilitated training and prediction for all ML algorithms except for the Cancerclass. From the training set, 10 models employing various ML techniques were developed. Afterward, 10 models were assessed on the validation set and contrasted the outcomes. The ultimate selection for the final FSS model was based on the top‐performing one and assessed by testing and in‐house sets.

### Establishment of a Predictive Model for ICI Response—Comparison between FSS and Published Signatures

To validate the predictive performance of FSS, it was compared against other previously documented ICI response signatures, including INFG_sig,^[^ ^21^ ^]^ Tcell_sig,^[^ ^21^ ^]^ Cytotoxic_sig,^[^ ^22^ ^]^ PDL1_sig,^[^ ^23^ ^]^ CAF_sig,^[^ ^24^ ^]^ and NLRP3_sig.^[^ ^25^ ^]^ The predictive value of FSS in ICI response prediction was evaluated against these published signatures in the testing sets. The methodologies for deriving the 6 mentioned signatures were based on their respective original research.

### Generation of FSS‐Related Prognostic Model—Cohorts

To further evaluate the FSS in survival prognostication, a cohort of 9815 TCGA patients across various cancers was randomly split into training (80%, *n* = 7865) and validation (20%, *n* = 1950) sets. Additionally, a testing set comprising 4434 patients from 10 cohorts and an in‐house set comprising 185 patients were enrolled for validation (Table S7, Supporting Information).

### Generation of FSS‐Related Prognostic Model—Model Training, Validation, and Testing

The prognostic value of FSS was investigated using SurvBenchmark,^[^ ^18^ ^]^ a benchmarking framework for survival models. This approach assesses both traditional methods and modern ML survival models. Initially, 15 algorithms in SurvBenchmark were employed in the study (Table S5, Supporting Information). The training set was utilized to train the model, while validation, testing and in‐house sets were employed for evaluation across 10 iterations of a fivefold CV. The final model was selected based on the optimal AUC and C‐index (incorporating Harrell's,^[^ ^75^ ^]^ Begg's,^[^ ^76^ ^]^ Uno's,^[^ ^77^ ^]^ and GH C‐index).^[^ ^78^ ^]^

### Senescence‐Associated β‐Galactosidase Assay

The activity of senescence‐associated β‐galactosidase (SA‐β‐gal) was assessed using a senescence detection kit (HY‐K1089, MCE) following the protocol provided by the manufacturer. SA‐β‐gal^+^ cells were quantified using a Nikon inverted microscope, and their percentage was calculated.

### Quantitative Real‐Time PCR (qRT‐PCR)

RNA samples were obtained using the TRIzol Reagent (Invitrogen, Carlsbad, CA). Subsequently, reverse transcription was executed using the HiScript III RT SuperMix (Vazyme, Nanjing, China). qRT‐PCR was facilitated by the AceQ Universal SYBR qPCR Master Mix (Vazyme). Primers are listed in Table S10 (Supporting Information).

### Western Blotting

Proteins from cell lysates were separated on 10% SDS‐PAGE gels and transferred onto polyvinylidene difluoride (PVDF) membranes (Merck Millipore, USA). Membranes were incubated overnight at 4 °C with primary antibodies, followed by treatment with secondary antibodies. Target protein signals were visualized using the Tanon Imaging System (China). The antibodies used are listed in Table S11 (Supporting Information).

### ELISA

The concentration of TGF‐β1 in the coculture medium was determined using the TGF‐β1 ELISA Kit (Abclonal) according to the manufacturer's instructions. Briefly, coculture medium samples were collected, centrifuged at 1000 × g for 10 min to remove cellular debris, and stored at −80 °C until analysis. Samples were added to ELISA plate wells pre‐coated with TGF‐β1 capture antibodies, followed by incubation at 37 °C. After multiple washing steps to remove unbound material, enzyme‐conjugated detection antibodies were applied, and substrate solution was added to develop the signal. The reaction was stopped with a stop solution, and the absorbance was measured at 450 nm using a microplate reader.

### Flow Cytometry

Cells underwent trypsin digestion, rinsed twice with staining buffer, and then subjected to staining utilizing Flow antibodies. For cytokine staining, cells were previously stimulated by Cell Stimulation Cocktail (ThermoFisher) for 4 h, and treated by Fixation/Permeabilization Kit (BD Biosciences) after surface staining. Subsequently, the stained cells were examined using a FACS Celesta multicolor flow cytometer (Becton Dickinson, Franklin Lakes, NJ), and the resulting data were analyzed utilizing the FlowJo software.

### IHC Analysis

The tissues underwent deparaffinization using xylene, followed by rehydration in an ethanol series. EDTA antigenic retrieval buffer (pH = 8) was then applied, and microwave treatment was utilized for antigenic retrieval. Afterward, a 3% methanol solution of hydrogen peroxide was used to counteract the intrinsic peroxidase activity. Subsequently, incubation with 1% albumin from goat serum was conducted to mitigate nonspecific adherence. The tissues were then subjected to overnight incubation with respective antibodies at 4 °C. Following washing, goat antimouse/rabbit IgG horseradish peroxidase polymer was applied to the tissue sections for 20 min. Chromogen 3, 3′‐Diaminobenzidine was employed for visualization.

### Tumor Xenograft Assay

C57BL/6 male mice aged 6 weeks were acquired from the Chinese Academy of Sciences in Shanghai and housed in a specific pathogen‐free facility. For the tumor model established through subcutaneous injection, KPC1199 (sh‐NC and sh‐CDC6) (5×10^5^ cells per site) transfected either with the anti‐PD1 (10 mg kg^−1^) or anti‐IgG (10 mg kg^−1^), were administered subcutaneously into the lateral abdomen of each mouse at day 9, day 12, and day 15. For the orthotopic model, cell suspensions of the specified cells into the pancreas of 6 week‐old male C57BL/6 mice to produce orthotopic tumors were injected. For subcutaneous tumors, tumor dimensions were recorded every 4 days for a duration of 25 days postinitial injection using the following formula: tumor volume (mm^3^) = 1/2 (a × b^2^), where “a” signifies the longest longitudinal diameter, and “b” represents the longest transverse diameter.

### HTVS

To identify potential CDC6 inhibitors, the 3D structure of CDC6 was downloaded from the AlphaFold database (AlphaFold ID: AF‐Q99741‐F1) and prepared using the Protein Preparation Wizard module, where hydrogen atoms were added, and energy minimization was performed with the OPLS2005 force field and a root mean square deviation (RMSD) cutoff of 0.30 Å. A receptor grid was then generated using the Receptor Grid Generation module, centered on ATP, with a grid box size of 20 × 20 × 20 Å. The HY‐L001P MCE Bioactive Compound Library, consisting of 23100 compounds in 2D format, was processed using the Schrödinger LigPrep module to add hydrogens, perform energy minimization, and generate 3D structures for virtual screening. Molecular docking was conducted using the Virtual Screening Workflow module, where compounds were screened with Glide in three stages: HTVS mode, followed by SP mode, and finally XP mode, with each stage refining the selection to the top 15% of compounds based on docking scores. A manual review of the top‐ranked compounds’ binding interactions and structures led to the selection of the top compounds for each of the two binding pockets, which were output as the final results.

### Ethics Approval and Consent of Participation

The studies were conducted in accordance with the Declaration of Helsinki and the International Conference on Harmonisation (ICH) Guidelines for Good Clinical Practice. Approval for the use of patient samples (No. 2021‐161) and animal experiments (No. RJ2024060) was granted by the Ethics Committee of Ruijin Hospital. Written informed consent was obtained from all participating patients.

### Statistical Analysis

All statistical analyses were conducted using R (version 4.2.2). Investigating the association between FSS and pathway‐specific scores or immunological characterization involved Spearman correlation analysis. Assessing disparities in survival was conducted through Kaplan–Meier curves coupled with the log‐rank test. Prognostic factors were determined via univariate Cox regression analysis. Comparisons between the two datasets utilized unpaired Student's *t*‐test for normally distributed variables and Mann–Whitney U‐test for non‐normally distributed ones. Statistical significance was attributed to a *p*‐value < 0.05 unless otherwise specified.

## Conflict of Interest

The authors declare no conflict of interest.

## Author Contributions

D.C., P.L., J.L., and L.Z. contributed equally to this work. Y.W. and H.L. conceived and supervised the study. D.C. performed the bioinformatics analysis. P.L. and J.L. conducted the experiments. D.C., P.L., J.L., and L.Z. analyzed the data. D.C. wrote the draft. D.C., P.L., J.L., L.Z., Y.L., S.Z., X.L., Y.W., and H.L. revised and validated the manuscript. All authors read and approved the final manuscript.

## Supporting information



Supporting Information

Supplemental Tables

## Data Availability

The data that support the findings of this study are available on request from the corresponding author. The data are not publicly available due to privacy or ethical restrictions. Essential scripts for the construction of FSS are available on the GitHub website (https://github.com/Dongjie‐orange/Pancancer_FSS).
