# Supplementary material for: A Distinguished Roadmap of Fibroblast Senescence in Predicting Immunotherapy Response and Prognosis Across Human Cancers
Source: Adv Sci (Weinh). 2024 Dec 30;12(7):2406624. doi: 10.1002/advs.202406624 (PMC11831569; doi:10.1002/advs.202406624)
Supplement: Supplementary file 1 — Supporting Information [file ADVS-12-2406624-s001.docx]

Supporting Information

A Distinguished Roadmap of Fibroblast Senescence in Predicting Immunotherapy Response and Prognosis across Human Cancers

Dongjie Chen, Pengyi Liu, Jiayu Lin, Longjun Zang, Yihao Liu, Shuyu Zhai, Xiongxiong Lu, Yuanchi Weng*, Hongzhe Li*

**Supplementary Figure 1**

**A**

**B**

**C**

**Figure S1.** **Cell-cell communication analysis using CellChat.** Circle plots show the cellular interaction weights and number of interactions between high-senescent (HS-CAF), low-senescent CAFs (LS-CAF) and other cell types in tumor microenvironment in cholangiocarcinoma (A), breast cancer (B) and colorectal cancer (C). Different colors in the circle plots represent different cell types and the edge width is proportional to the indicated cell-cell interaction weights.

**Supplementary Figure 2**

**Figure S2.** **FSS scores across pan-cancer TCGA cohorts.**

**Supplementary Figure 3**

**
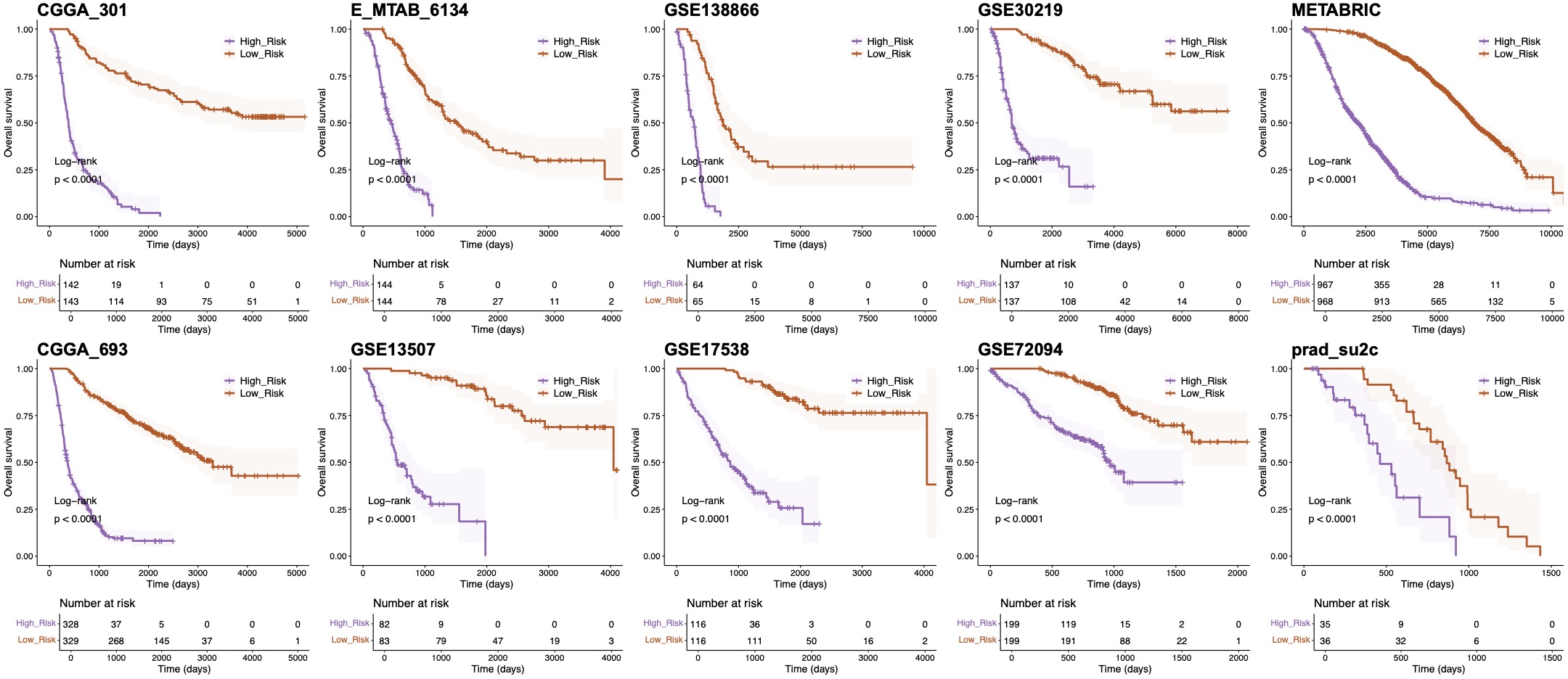
**

**Figure S3.** **Kaplan‑Meier curves comparing OS between High‑risk and Low‑risk patients in each testing set.**

**Supplementary Figure 4**

**Figure S4.** **The association between FSS-related risk score and OS among each TCGA pan-cancer cohort.﻿﻿**

**Supplementary Figure 5**

**A**

**B**

**Figure S5.** **﻿﻿Effectiveness assessment of FSS-derived nomogram features in predicting pan-cancer prognosis.** (A) Nomogram showing the prediction for the overall survival of patients in the pan-cancer TCGA cohorts. (B) ﻿The calibration plot showing the consistency between the prediction of FSS and real survival.

**Supplementary Figure 6**

**
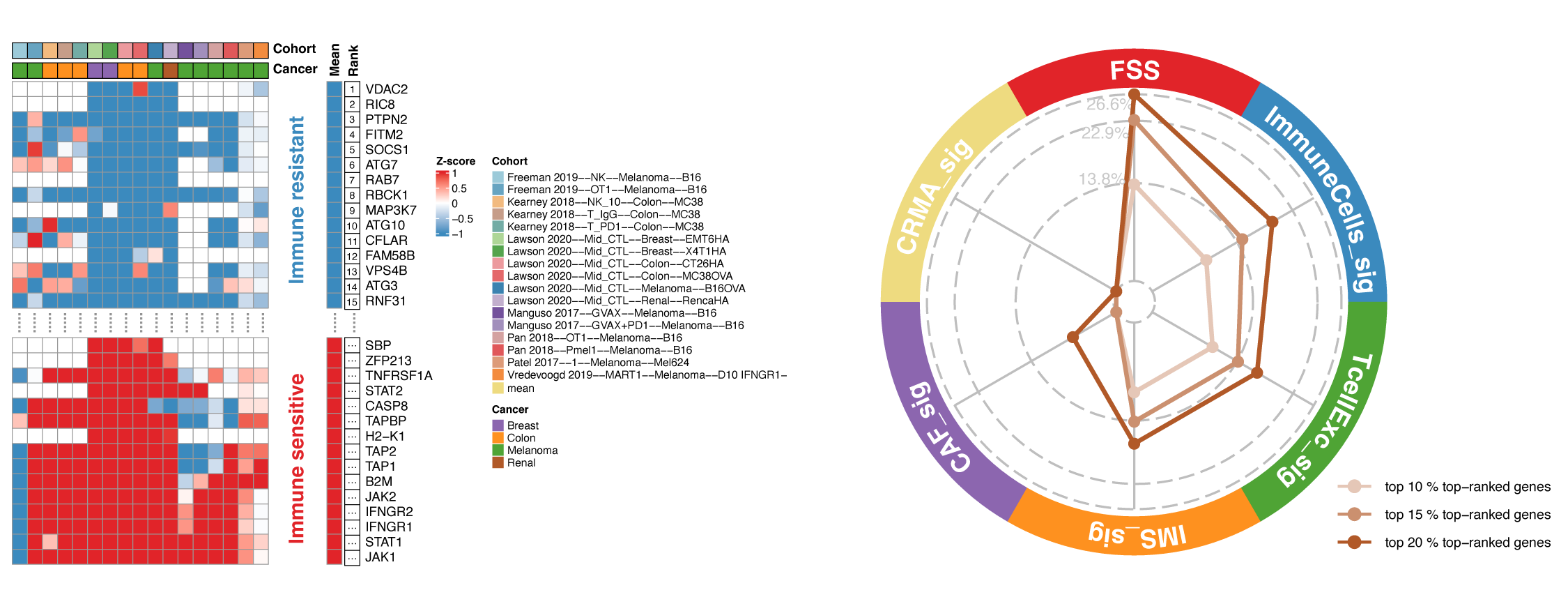
**

**B**

**A**

**Figure S6.** **﻿﻿CRISPR/Cas9 screening of CDC6.** (A) Assessing the ranking of genes by their impact on anti-cancer immune response across 17 CRISPR/Cas9 datasets. (B) Radar plot depicting the percentage of top‑ranked genes for FSS and other predictive signatures.

**Supplementary Figure 7**

**Figure S7.** **﻿﻿Heatmap showing the correlation between expression of CDC6 and OS among pan-cancer TCGA cohorts.**

**Supplementary Figure 8**


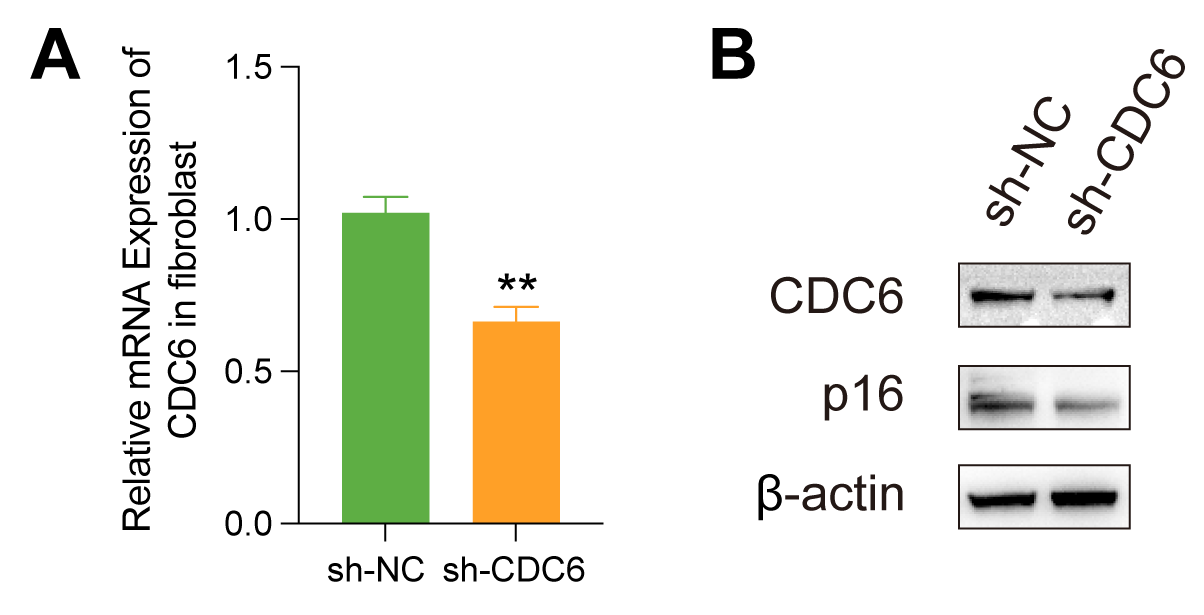


**Figure S8.** **﻿﻿Detection of CDC6 expression in fibroblasts within sh-CDC6 PC organoids.** (A) mRNA expression of CDC6 in fibroblasts within sh-CDC6 PC organoids. (B) Protein expression of CDC6 and p16 in fibroblasts within sh-CDC6 PC organoids.
